# Supplementary material for: Transporter-mediated depletion of extracellular proline directly contributes to plant pattern-triggered immunity against a bacterial pathogen
Source: Nat Commun. 2024 Aug 15;15:7048. doi: 10.1038/s41467-024-51244-6 (PMC11327374; doi:10.1038/s41467-024-51244-6)
Supplement: Supplementary file 1 — Supplementary Information [file 41467_2024_51244_MOESM1_ESM.pdf]

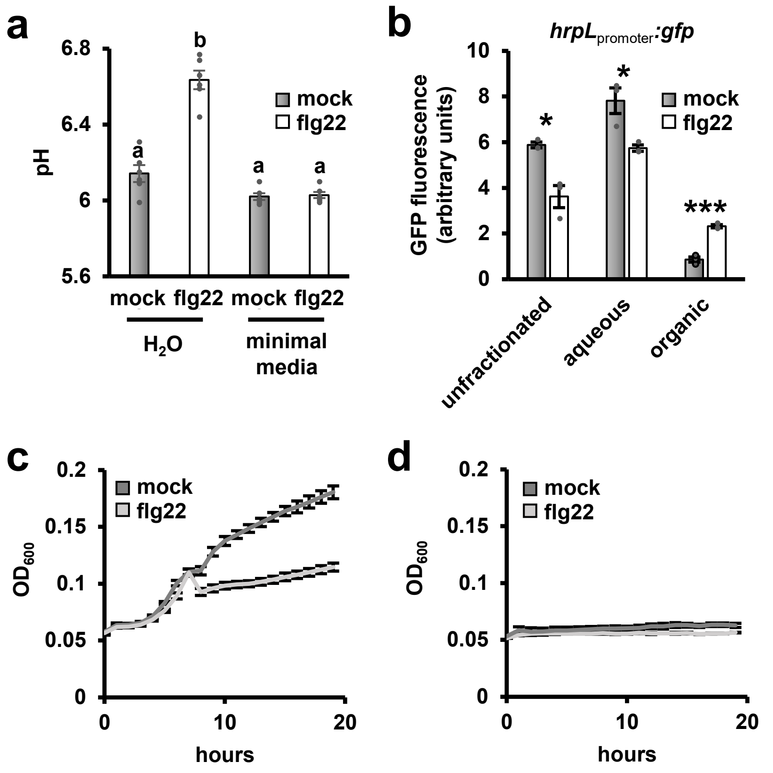

Supplementary Fig. 1. Impacts of pH buffering and organic solvent extraction on the T3SS- and growth-inducing bioactivity of apoplastic washing fluid (AWF). AWF was extracted from leaves treated with 100 nM flg22 or a mock treatment for six hours. **a,** Graphed are means ± SE of pH measurements of isolated AWF resuspended in either H_2O or phosphate buffered minimal medium, n = 6. Lower case letters denote significance groupings based on ANOVA with Tukey's HSD, $p < 0.01$. Data shown were pooled from two independent experiments. **b-d,** Chloroform was added to AWF, and the resulting aqueous and organic phases were lyophilized to dryness, resuspended in water, and tested for *P. syringae* type III secretion- and growth-inducing activity. (b) Graphed are means ± SE of GFP fluorescence from a DC3000 $hrpL_{\text{promoter}}$:*gfp* strain cultured in unfractionated AWF, AWF aqueous phase or AWF organic phase, n = 3. Fluorescence values were normalized to fluorescence from an empty vector strain under identical treatment conditions. Data are representative of two independent experiments. Asterisks denote significance based on two-sided *t*-test between mock and flg22, * is $p < 0.05$, *** is $p < 0.001$. (c,d) Timecourse of DC3000 $hrpL_{promoter}$:*gfp* growth in (c) aqueous- or (d) organic-extracted AWF. Graphed are means ± SE of culture optical density at λ = 600 nm (OD_{600}) measurements, n = 3. Data are representative of two independent experiments.

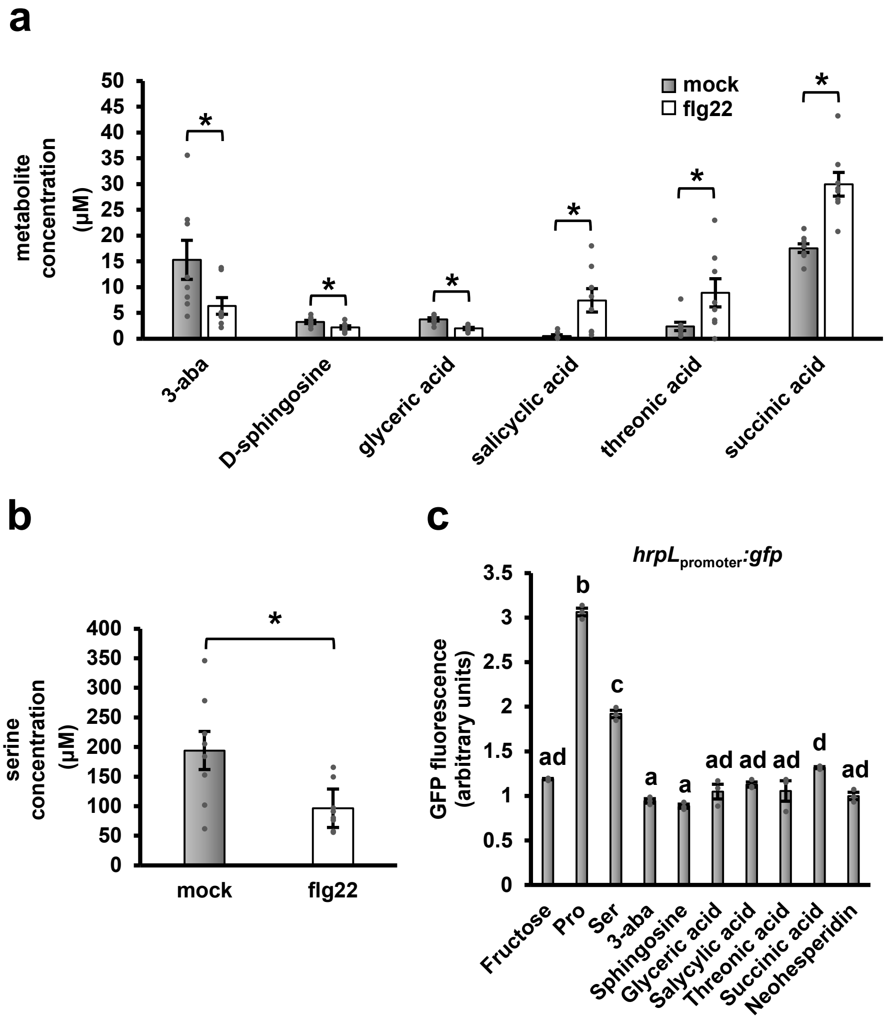

Supplementary Fig. 2. Abundance and type III secretion system-inducing activity of metabolites that differentially accumulate in the apoplast of flg22 treated plants. (a,b) Absolute quantification of metabolites that significantly differed in apoplastic wash fluid (AWF) from flg22 treated plants. Graphed are means of concentrations (μM) for each metabolite. Error bars are ± SE, n = 8. Asterisks denote statistical significance based on *t*-test, * is $p < 0.05$. Results are from eight independent experiments. **(c)** DC3000 carrying a $hrpL_{promoter}$:*gfp* or empty vector control plasmid were cultured for six hours in minimal medium with 10 mM fructose and the following: 100 μM serine, 100 μM proline, 30 μM succinic acid, 15 μM 3-aba (3-aminoisobutyric acid), 10 μM threonic acid, 10 μM salicylic acid, 5 μM glyceric acid or 5 μM sphingosine. Concentrations of metabolites were selected to closely match the difference in abundance between mock and flg22 samples shown in panels a and b. Graphed are means ± SE of normalized GFP fluorescence, n = 3. Data are representative of two independent experiments. Lower case letters denote significance groupings based on ANOVA with Tukey's HSD, $p < 0.01$.

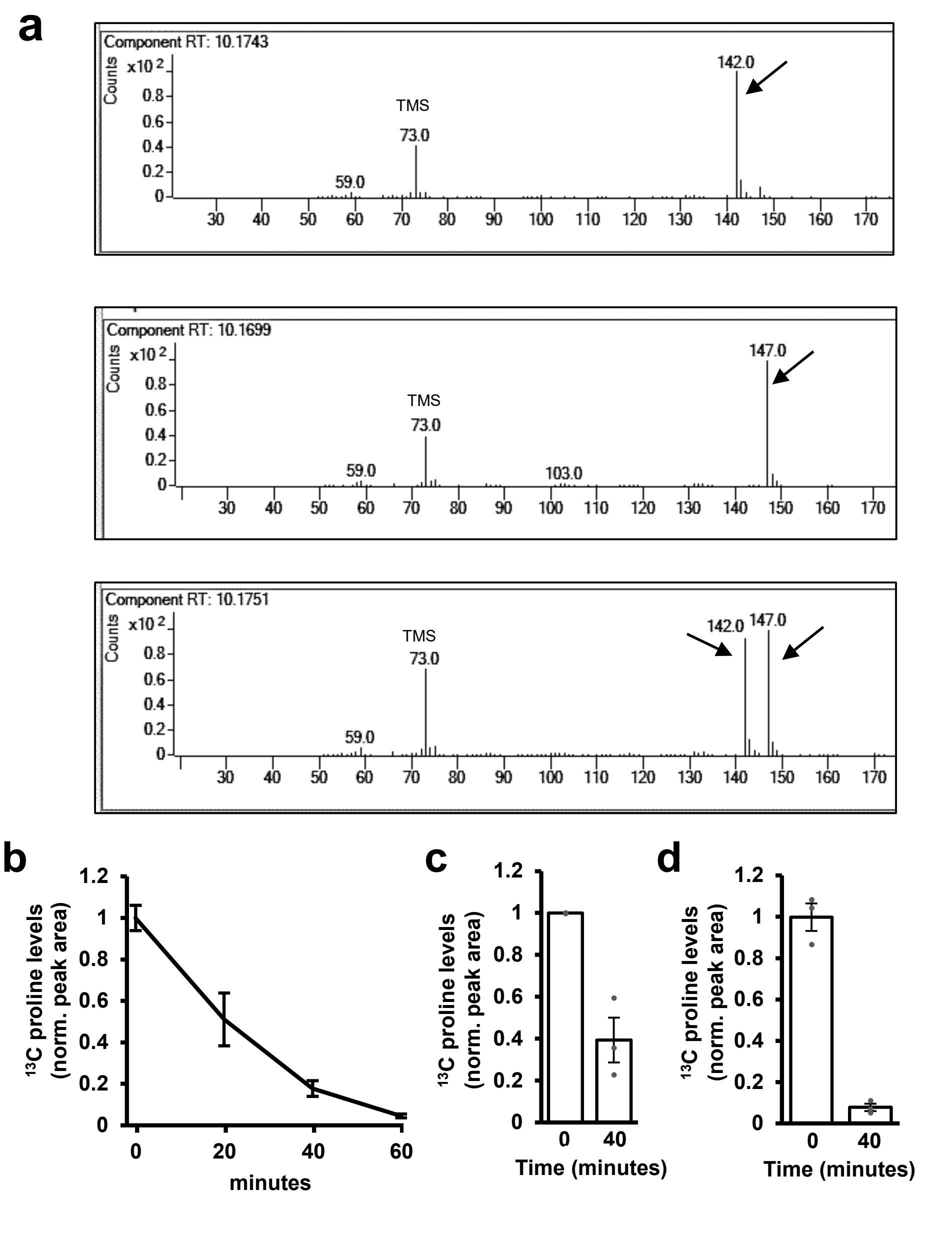

Supplementary Fig. 3. ^{13}C L-proline can be detected in apoplastic wash fluid (AWF) isolated from Arabidopsis leaves and is rapidly removed from the apoplast of detached leaves. a, GC-MS spectra of ^{12}C L-proline (upper panel), ^{13}C L-proline (middle panel), or a mixture of both ^{12}C L-proline and ^{13}C L-proline (lower panel). Shown are the mass spectra collected at a retention time of 10.17 seconds. The arrows indicate the base peaks of ^{12}C L-proline or ^{13}C L-proline that were used to calculate their respective relative abundances. The shared peak at 73.0 is likely the trimethylsilyl (TMS) group added by chemical derivatization. **b,** A solution of 500 μM ^{13}C L-proline was infiltrated into Arabidopsis leaves. Immediately after infiltration the infiltrated leaves were excised from the plant and placed in a humid chamber. At the indicated time points, AWF was extracted from the detached leaves and analyzed by GC-MS. Data shown are average normalized peak areas ± SE of ^{13}C L-proline in AWF, n = 2. Results shown are from two independent experiments. **c-d,** Arabidopsis *prot2* (c) and *lht1* (d) leaves were infiltrated with water. Eight hours later the same leaves were infiltrated with 500 μM ^{13}C-proline and 164 μM ribitol. Apoplastic wash fluid (AWF) was isolated from treated leaves at time points indicated, and the abundance of ^{13}C-proline and ribitol measured by GC-MS. Graphed are means ± SE of ^{13}C-proline normalized to ribitol and scaled to a value of 1 at T=0, n = 3. Data are from three independent experiments.

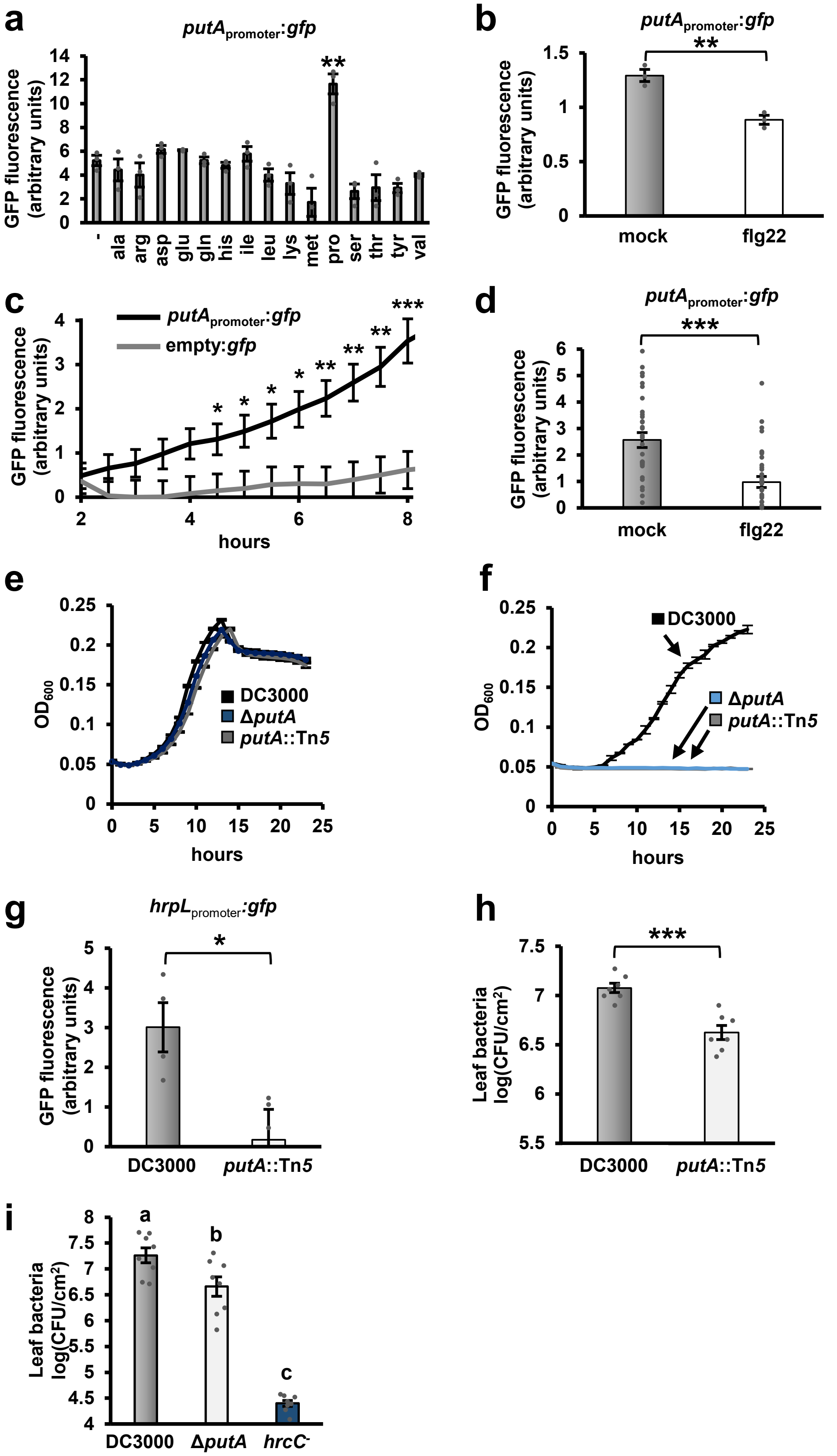
a
putA promoter:gfp
GFP fluorescence (arbitrary units)
14
12
10
8
6
4
2
0
**
-
ala
arg
asp
glu
gln
his
ile
leu
lys
met
pro
ser
thr
tyr
val
b
putA promoter:gfp
**
1.5
1
0.5
0
mock
flg22
c
putA promoter:gfp
empty:gfp
*
**
***
hours
2
4
6
8
d
putA promoter:gfp
***
mock
flg22
e
OD600
0.25
0.2
0.15
0.1
0.05
0
DC3000
ΔputA
putA::Tn5
hours
0
5
10
15
20
25
f
DC3000
ΔputA
putA::Tn5
hours
g
hrpL promoter:gfp
*
DC3000
putA::Tn5
h
Leaf bacteria log(CFU/cm²)
***
7.5
7
6.5
6
5.5
DC3000
putA::Tn5
i
Leaf bacteria log(CFU/cm²)
a
b
c
8
7.5
7
6.5
6
5.5
5
4.5
4
DC3000
ΔputA
hrcC-

Supplementary Fig. 4. DC3000 *putA* is required for proline-induced growth, T3SS deployment and virulence. a, DC3000 carrying either a $putA_{promoter}$:*gfp* reporter plasmid or empty *gfp* plasmid were incubated for six hours in minimal medium (MM) supplemented with or without 10 mM of an amino acid as indicated. Graphed are means ± SE of normalized GFP fluorescence from $putA_{promoter}$:*gfp* cultures, n = 3. **b,** DC3000 carrying either a $putA_{promoter}$:*gfp* reporter plasmid or empty *gfp* plasmid were incubated in apoplastic wash fluid isolated from flg22 or mock-treated Arabidopsis leaves for five hours. Graphed are means ± SE of normalized GFP fluorescence from $putA_{promoter}$:*gfp* cultures, n = 3. Data are from three independent experiments. **c,** DC3000 carrying either a $putA_{promoter}$:*gfp* reporter plasmid or empty *gfp* plasmid were syringe-infiltrated into Arabidopsis leaf tissue**.** Graphed are means ± SE of GFP fluorescence from infected tissue, n = 24 for $putA_{promoter}$:*gfp* and n = 12 for empty:*gfp*. **d,** DC3000 carrying either a $putA_{promoter}$:*gfp* reporter plasmid or empty *gfp* plasmid were syringe-infiltrated into Arabidopsis leaf tissue pre-treated with 100 nM flg22 or a mock treatment for 18 hours. Graphed are means ± SE of normalized GFP fluorescence from infected tissue six hours after infection, n = 36. **e-f,** Time course of DC3000, Δ*putA* or *putA*::Tn*5* growth in M9 medium supplemented with 10 mM (E) glutamate or (F) proline. Graphed are means ± SE of culture optical density at λ=600 nm (OD_{600}) measurements at the indicated time points, n = 3. **g,** Graphed are means ± SE of normalized GFP fluorescence from DC3000 $hrpL_{\mathrm{promoter}}$:*gfp* or *putA*::Tn*5* $hrpL_{\mathrm{promoter}}$:*gfp* strains cultured in MM supplemented with or without 200 µM proline, n = 3. Data shown are representative of two independent experiments. **h,** 2 x 10^6 cfu/mL of DC3000 or DC3000 *putA*::Tn*5* was infiltrated into Arabidopsis leaves. Leaf bacteria were enumerated on day 3 by serial dilution plating of leaf extracts. Graphed are log-transformed means ± SE of colony-forming units (cfu) isolated from infected tissue. n = 7. **i,** 2 x 10^6 cfu/mL of DC3000, DC3000 Δ*putA* or DC3000 $hrcC^-$ was infiltrated into Col-0 and *prot2-3* leaves. Leaf bacteria were enumerated by serial dilution plating of leaf extracts. Graphed are log transformed means ± SE of colony-forming units (cfu), n = 8. Lower case letters in panel i denote significance groupings based on ANOVA with Tukey's HSD, $p < 0.05$. Asterisks in panels a-d, g and h denote statistical significance based on two-sided *t*-test, * is $p < 0.05$, ** is $p < 0.01$, *** is $p < 0.001$. Results in panels a and c-g are representative of two independent experiments. Data in h-i are from two independent experiments.

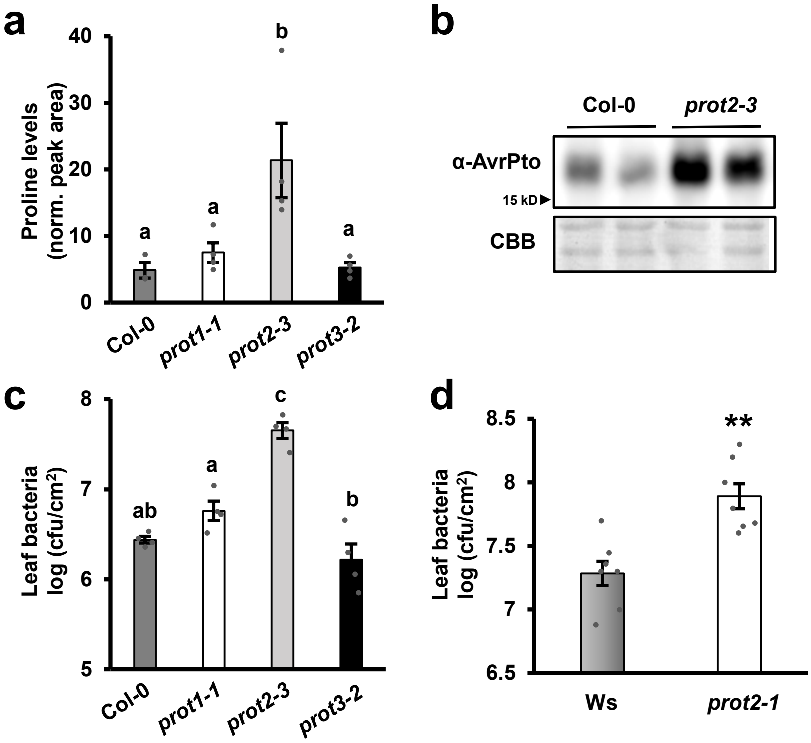

Supplementary Fig. 5. An Arabidopsis *prot2* mutant accumulates higher levels of apoplastic proline and is more susceptible to *P. syringae* DC3000 infection. a, GC-MS measurements of proline levels in apoplastic wash fluid (AWF) isolated from wild-type Col-0, *prot1-1, prot2-3,* or *prot3-2* leaves. Graphed are means ± SE of the peak area of proline normalized to the internal ribitol standard included in the extraction solution, n = 4. Each sample is AWF isolated from leaves of one plant. Data are from four independent experiments**.** Lower case letters denote statistical groupings based on ANOVA with Tukey's HSD, $p < 0.05$. **b,** AvrPto protein abundance in leaf tissue five hours after infiltration of Col-0 or *prot2-3* leaves with DC3000. Upper panel is immunoblot detection of AvrPto levels in protein extracts from infected leaves, lower panel is Coomassie Brilliant Blue (CBB) staining of the immunoblot to assess equal loading. Results are representative of two independent experiments. **c,** 2 x 10^6 cfu/mL of DC3000 was infiltrated into leaves of Col-0, *prot1-1, prot2-3,* or *prot3-2*. Leaf bacteria were enumerated on day 3 by serial dilution plating of leaf extracts. Graphed are log-transformed means ± SE of colony-forming units (cfu) of bacteria isolated from infected tissue, n = 4. Data shown are representative of two independent experiments**.** Lower case letters denote statistical groupings based on ANOVA with Tukey's HSD, $p < 0.05$. **d,** 2 x 10^6 cfu/mL of DC3000 was infiltrated into leaves of wild-type Wassilewskija (Ws) or *prot2-1*(Ws) plants. Leaf bacteria were enumerated on day 3 by serial dilution plating of leaf extracts. Graphed are log-transformed means ± SE of cfu of bacteria isolated from infected tissue, n = 8. Asterisks denote significance based on *t*-test between Ws and *prot2-1*(Ws), ** is $p < 0.01$. Data are from two independent experiments.

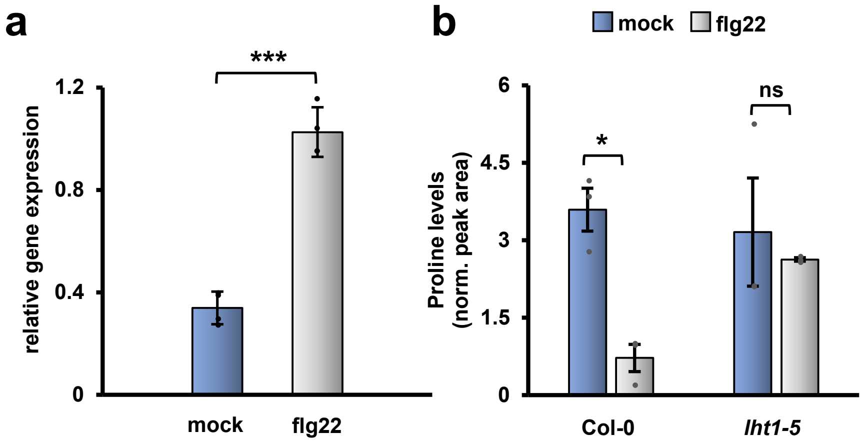

Supplementary Fig. 6. *LHT1* is induced by flg22 treatment and apoplastic proline levels are not altered by flg22 treatment in *lht1-5* leaves. a, Leaves of four-week-old Col-0 leaves were infiltrated with 100 nM flg22 or a mock treatment for 40 minutes. *LHT1* transcripts were measured by RT-qPCR. Graphed are means ± SE, n = 4. Data are representative of two independent experiments. **b,** Proline levels in apoplastic wash fluid (AWF) isolated from Col-0 and *lht1-5* leaves eight hours after syringe-infiltration with 100 nM flg22 or a mock treatment. Graphed are means ± SE of normalized peak areas for proline, n = 3. Asterisks in both panels denote significance based on two-sample *t*-test, * is $p < 0.05$, *** is $p < .0001$, ns is $p > 0.05$.

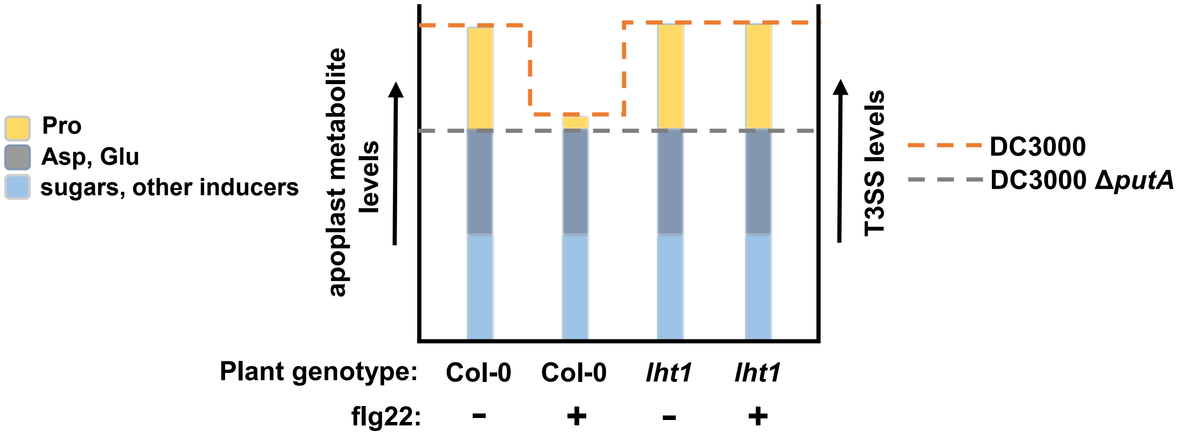

Supplementary Fig. 7. Hypothetical model of how metabolites in the Arabidopsis leaf apoplast quantitatively contribute to induction of T3SS genes in DC3000. Within the Arabidopsis leaf apoplast, DC3000 encounters a mixture of T3SS-inducing metabolites. Shown is a graphical model that describes how flg22 treatment and the presence/absence of LHT1 alter the abundance of T3SS-inducing metabolites in the apoplast (bar graph), and how these changes impact the levels of T3SS induction in DC3000 and DC3000 Δ*putA* (dashed lines).
